# Altered left atrial 4D flow characteristics in patients with paroxysmal atrial fibrillation in the absence of apparent remodeling

**DOI:** 10.1038/s41598-021-85176-8

**Published:** 2021-03-16

**Authors:** Ahmet Demirkiran, Raquel P. Amier, Mark B. M. Hofman, Rob J. van der Geest, Lourens F. H. J. Robbers, Luuk H. G. A. Hopman, Mark J. Mulder, Peter van de Ven, Cornelis P. Allaart, Albert C. van Rossum, Marco J. W. Götte, Robin Nijveldt

**Affiliations:** 1 Department of Cardiology, Amsterdam UMC, Amsterdam Cardiovascular Sciences, Vrije Universiteit Amsterdam, De Boelelaan 1117, 1081 HV Amsterdam, The Netherlands; 2 Department of Radiology and Nuclear Medicine, Amsterdam UMC, Vrije Universiteit Amsterdam, Amsterdam, The Netherlands; 3 Department of Radiology, Division of Image Processing, Leiden University Medical Center, Leiden, The Netherlands; 4 Department of Epidemiology and Biostatistics, Amsterdam UMC, Vrije Universiteit Amsterdam, Amsterdam, The Netherlands; 5 Department of Cardiology, Radboud University Medical Center, Geert Grooteplein Zuid 10, 6525 GA Nijmegen, The Netherlands

**Keywords:** Arrhythmias, Thromboembolism

## Abstract

The pathophysiology behind thrombus formation in paroxysmal atrial fibrillation (AF) patients is very complex. This can be due to left atrial (LA) flow changes, remodeling, or both. We investigated differences for cardiovascular magnetic resonance (CMR)-derived LA 4D flow and remodeling characteristics between paroxysmal AF patients and patients without cardiac disease. In this proof-of-concept study, the 4D flow data were acquired in 10 patients with paroxysmal AF (age = 61 ± 8 years) and 5 age/gender matched controls (age = 56 ± 1 years) during sinus rhythm. The following LA and LA appendage flow parameters were obtained: flow velocity (mean, peak), stasis defined as the relative volume with velocities < 10 cm/s, and kinetic energy (KE). Furthermore, LA global strain values were derived from b-SSFP cine images using dedicated CMR feature-tracking software. Even in sinus rhythm, LA mean and peak flow velocities over the entire cardiac cycle were significantly lower in paroxysmal AF patients compared to controls [(13.1 ± 2.4 cm/s vs. 16.7 ± 2.1 cm/s, *p* = 0.01) and (19.3 ± 4.7 cm/s vs. 26.8 ± 5.5 cm/s, *p* = 0.02), respectively]. Moreover, paroxysmal AF patients expressed more stasis of blood than controls both in the LA (43.2 ± 10.8% vs. 27.8 ± 7.9%, *p* = 0.01) and in the LA appendage (73.3 ± 5.7% vs. 52.8 ± 16.2%, *p* = 0.04). With respect to energetics, paroxysmal AF patients demonstrated lower mean and peak KE values (indexed to maximum LA volume) than controls. No significant differences were observed for LA volume, function, and strain parameters between the groups. Global LA flow dynamics in paroxysmal AF patients appear to be impaired including mean/peak flow velocity, stasis fraction, and KE, partly independent of LA remodeling. This pathophysiological flow pattern may be of clinical value to explain the increased incidence of thromboembolic events in paroxysmal AF patients, in the absence of actual AF or LA remodeling.

## Introduction

Atrial fibrillation (AF), the most common arrhythmia, is associated with a 4–5 fold increased risk of thromboembolic events^1,2^. Clinical evidence suggests that the risk of thromboembolic events is not different between patients with paroxysmal AF and those with sustained AF^3,4^. The thromboembolic events in AF patients are mainly attributed to embolism of thrombus from the left atrium. However, existing stroke risk-scoring systems (e.g., CHA_2_DS_2_-VASc) in patients with AF are merely based on demographic and clinical risk factors, disregarding individual flow characteristics in the left atrium^5^. Recently, it was demonstrated that left atrial (LA) blood flow velocities are noticeably reduced in patients with sinus rhythm and a history of AF^6^. Furthermore, in patients with subclinical AF, no clear temporal relation was found between the occurrence of stroke and episodes of AF^7^. These observations suggest that reduced LA global flow may persist during sinus rhythm and therefore may increase the risk of thromboembolic events, even in the absence of AF.

Since AF is believed to be a progressive disease leading to atrial dilation and mechanical dysfunction of the left atrium over time^8,9^, structural and functional remodeling may also play a role in the occurrence of thromboembolic events in AF patients^10,11^. The interplay between LA remodeling and flow patterns, however, is poorly characterized. Therefore, a better understanding of LA remodeling and flow characteristics in patients with paroxysmal AF is warranted.

Cardiovascular magnetic resonance (CMR) not only allows detailed assessment of atrial remodeling but also four-dimensional (4D) flow analysis^12^. This 4D flow analysis enables an elaborate description of intra-atrial 3-directional flow velocities during the cardiac cycle and its derivatives, including stasis fraction and kinetic energy (KE). Combined with CMR feature tracking^13^, 4D flow analysis may provide new insights into atrial hemodynamics in paroxysmal AF, leading to a better understanding of mechanisms of reduced LA blood flow.

For this purpose, in this proof-of-concept study, CMR-derived LA 4D flow characteristics and remodeling parameters were compared between patients with a history of paroxysmal AF and patients without cardiac disease.

## Methods

This single-center study was conducted according to the principles outlined in the 1964 Declaration of Helsinki and its later amendments. Collection and management of data was approved by the local medical ethics committee (Amsterdam UMC, Vrije Universiteit Amsterdam, the Netherlands). Written informed consent was obtained from all individual participants included in the study.

### Study population

Ten patients with a history of paroxysmal AF (3 female, age = 61 ± 8 years) and 5 age/gender matched controls (1 female, age = 56 ± 1 years) underwent CMR examination during sinus rhythm. Patients with paroxysmal AF scheduled for a pulmonary vein isolation procedure were consecutively enrolled. Patients who had undergone an electrical or medical cardioversion procedure in the 6 weeks prior to CMR scanning were excluded. Other exclusion criteria were significant renal impairment (defined as eGFR < 30 ml/min/kg), left ventricular ejection fraction < 50%, clinically significant valvular disease (mild to severe), and a contra-indication for CMR (e.g., implanted devices, claustrophobia). Stroke risk evaluation was performed using the CHA_2_DS_2_-VASc score, which is the current recommended risk model according to the guidelines.

### CMR protocol

**Figure 1:**
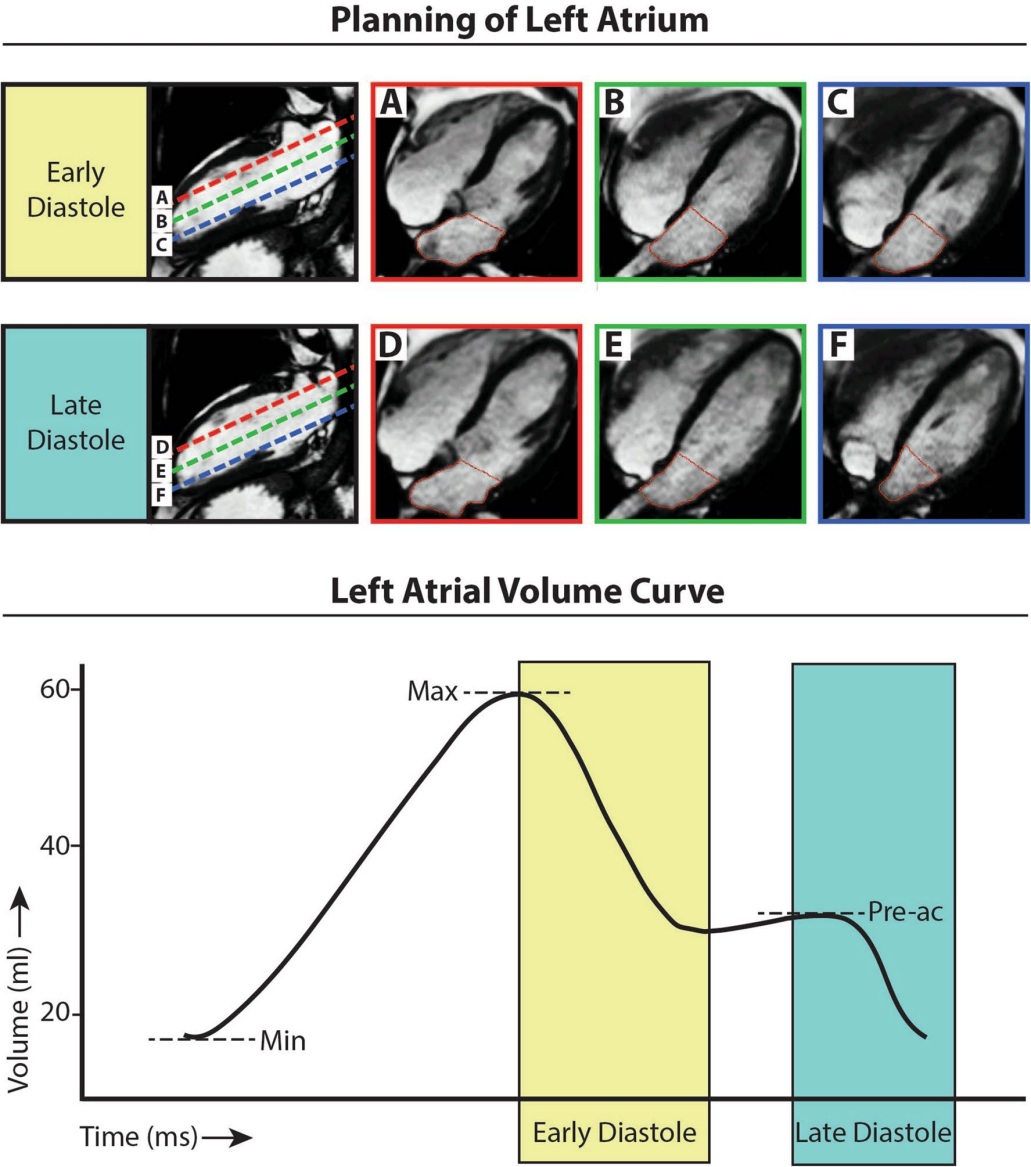
A representative example of planning and tracing of the left atrial cine images. Image planes are orientated parallel to the 4 chamber cine image as demonstrated on the 2-chamber long axis images Based on the contour tracings (**A**–**F**) the left atrial volume curve over time (lower panel) is derived. (MASS version 2017-Exp, Leiden University Medical Center, Leiden, the Netherlands; Adobe Illustrator CS6, version 16.0.0, https://www.adobe.com/products/illustrator.html). Abbreviations: AF, atrial fibrillation; min, minimum; max, maximum; pre-ac, before atrial contraction; ml, milliliter; ms, millisecond.

All CMR examinations were performed on a clinical 1.5 T MRI system (Magnetom Avanto, Siemens, Erlangen, Germany). An ECG-gated, balanced steady-state free precession (b-SSFP) sequence was performed for the acquisition of long-axis cine imaging including 4-chamber, 3-chamber, and 2-chamber views. Typical parameters were: in-plane spatial resolution 1.8 × 1.3 mm^2^, slice thickness/slice gap 5/5 mm, flip angle 60°, temporal resolution < 50 ms, repetition time/echo time 3.1/1.6 ms. A consecutive stack of 14–16 slices was planned parallel to the 4-chamber cine image covering the complete volume of the left atrium (Fig. 1). Each cine image series was acquired during 1 breath-hold at mild expiration. For the assessment of LA blood flow velocity and flow-derived hemodynamic parameters (e.g., stasis and kinetic energy), prospective ECG-gated, 3-dimensional phase-contrast imaging with 3-directional velocity encoding (4D flow CMR) was used^14^. Global blood flow velocity data were acquired within the left atrium using a single, free-breathing acquisition. 4D flow CMR pulse sequence parameters were as follows: in-plane spatial resolution 3.1 × 3.1mm^2^, slice thickness 3 mm, flip angle 15°, temporal resolution < 32 ms, GRAPPA factor 2. The velocity sensitivity was set to 100 cm/s and the total acquisition time was 15–20 min, depending on the heart rate.

### Volume and function analysis

Images were analyzed using dedicated research software (MASS version 2017-Exp, Leiden University Medical Center, Leiden, the Netherlands). For LA volume analysis, endocardial contours were manually traced on all LA slices in every phase of the cardiac cycle. The pulmonary veins, LA appendage, and coronary sinus were excluded. A straight line was drawn between the leading edges of the mitral valve annulus to separate the left atrium from the left ventricle (Fig. 1). From all LA volumes obtained throughout the cardiac cycle, the following parameters were derived: maximum LA volume, LA volume before atrial contraction, and minimum LA volume (Fig. 1). From these parameters, reservoir LA volume, LA conduit volume, and LA active emptying volume were calculated (Table 1)^15^. The contribution of these fractional LA volume changes to LA function was separately analyzed as total LA function, passive LA function, and booster LA function (Table 1). The LA appendage was separately traced on all LA slices and the respective volume and function parameters were analyzed in a similar fashion. LV volume, function, and mass analysis were performed using the biplane area-length method^16^. For this purpose, endocardial and epicardial contours were manually traced at end-diastole and end-systole in the 2- and 4-chamber views.

**Table 1:** Calculation of left atrial volumes and function.

	Equation
**LA volume**	
Reservoir LA volume	Max LA volume–min LA volume
LA conduit volume	Max LA volume–LA volume pre-ac
LA active emptying volume	LA volume pre-ac–min LA volume
**LA function**	
Total LA function	Max LA volume–min LA volume × 100/max LA volume
Passive LA function	Max LA volume–LA volume pre-ac × 100/max LA volume
Booster LA function	LA volume pre-ac–min LA volume × 100/LA volume pre-ac

Abbreviations: *LA* left atrial, *max* maximum, *min* minimum, *pre-ac* before atrial contraction.

### 4D flow data analysis

**Figure 2:**
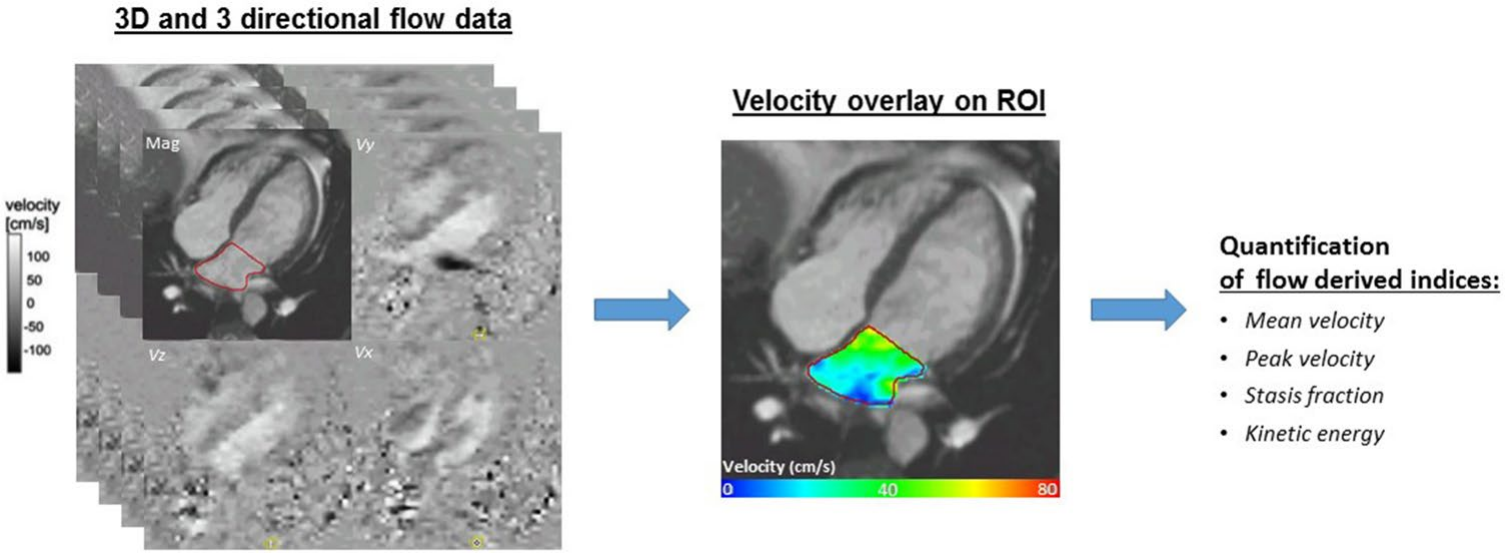
Illustration of the 4D flow CMR acquisition and analysis process. On the left panel, the 3-dimensional and 3-directional acquisition process is displayed through flow velocity and magnitude images. In the middle panel, velocity overlay on the left atrium during early diastole is shown. On the right panel, flow characteristics which were generated through 4D flow CMR data are displayed. (MASS version 2017-Exp, Leiden University Medical Center, Leiden, the Netherlands; Microsoft, PowerPoint 2016, https://www.microsoft.com/en/microsoft-365/powerpoint). Abbreviations: 3D, 3 dimensional; ROI, region of interest; cm/s, centimeter per second.

All 4D flow data were corrected for Maxwell terms in the image reconstruction at the scanner^17^. Further analysis was performed using dedicated research software (MASS version 2017-Exp, Leiden University Medical Center, Leiden, the Netherlands). The remaining velocity offset was corrected using a spatial linear interpolation of the offset assessed in the stationary tissue. 4D flow data were co-registered with the cine images covering the whole left atrium to guide anatomic LA orientation (Fig. 2). Mean and peak flow velocity data were obtained using velocity–time curves which display spatial average velocities within the defined region of interest. Using the cine images and 2-dimensional mitral inflow assessment, specific time periods (systole, diastole) and points (peak E wave, peak A wave) were identified. For each subject, LA and LA appendage mean and peak flow velocities were calculated for specified time parameters (during systole, diastole, at peak E wave, at peak A wave). Stasis is defined as the volume fraction with a flow velocity below 10 cm/s during the entire cardiac cycle within the region of interest (LA, LA appendage). This was assessed as the percentage of voxels. For each voxel, kinetic energy (KE) was computed by multiplying 0.5 times the density of blood (1.025 g/mL) with the voxel volume representing the mass, and the 3-directional velocity magnitude squared and was expressed as mJ. The KE in the region of interest was computed by summation of the KE over all voxels. LA and LA appendage mean and peak KE were quantified for all time parameters (during systole, diastole, at peak E wave, at peak A wave) and indexed to the maximum LA volume.

### Feature tracking strain analyses

Myocardial feature tracking analysis was performed on the b-SSFP cine images using dedicated commercial software (Circle Cardiovascular Imaging, Inc, Calgary, Canada). All LA contours were manually traced in the 2- and 4-chamber cine views at the end-diastolic phase. Accurate tracking of the atrial wall was ensured by visually examining tracking performance and manually adjusting the contours when needed. To improve tracking, pulmonary veins and the LA appendage were excluded from the analysis. Average global longitudinal LA strain and strain rate profiles were assessed. In line with segmented LA function analysis, the atrial strain was divided into 3 phases: reservoir, conduit, and active emptying. From the strain curves, 3 strain rate parameters were derived: peak positive, peak early negative, peak late negative. Finally, LV global longitudinal strain analysis was performed following standard assessment.

All CMR contour tracings including volume/function, 4D flow CMR, and feature tracking were performed by an EACVI level III certified CMR expert (A.D.) and controlled by an EACVI level III certified CMR expert (R.N.) with over 15 years of CMR experience. To define inter-observer variability, a second independent observer (L.H.), blinded to the other reader's results re-analyzed the LA 4D flow and feature tracking strain data through the application of a second 3D segmentation of the LA. These tests of reproducibility were represented by the intra-class correlation coefficient.

### Statistical analysis

Continuous variables are presented as mean ± standard deviation or median with interquartile range. Comparisons between two groups were made with the independent samples T-test for normally distributed data or Mann–Whitney U test for non‐parametric data. Flow differences between the LA and LA appendage within the groups were compared with a paired sample T-test. Categorical variables are summarized by frequency (percentage) and compared between two groups using a Fisher's exact test. Association between continuous variables was quantified by Spearman’s correlation. The two-sided significance level was set at 5%. As this was an explorative study correction for multiple testing was not performed. Statistical analysis was performed using the Statistical Package for Social Sciences software (IBM SPSS statistics 26).

## Results

Characteristics of the study population are summarized in Table 2. The 4D flow data of one subject with paroxysmal AF could not be analyzed, due to damage in the data reconstruction process of the large data file. Patients with paroxysmal atrial fibrillation (n = 10) and controls (n = 5) were comparable for age, gender, and body mass index. In the group of patients with paroxysmal AF, 5 patients had a CHA_2_DS_2_-VASc risk-score of 1, while only 1 patient had a CHA_2_DS_2_-VASc risk-score of 2. The remaining patients had a CHA_2_DS_2_-VASc risk-score of 0. Five patients with paroxysmal AF had a history of a planned electrical cardioversion. The time period between the cardioversion and enrolment in the study varied between 22 and 46 months. While controls had neither acetylsalicylic acid nor oral anticoagulant therapy, 2 patients with paroxysmal AF were on vitamin K-antagonist treatment and 3 patients were on novel oral anticoagulants. Finally, no significant differences were observed regarding lipid levels, blood cell counts (e,g., RBC), and coagulation indexes (e.g., INR) between the groups (Supplementary file).

**Table 2:** Patient characteristics.

	Controls (n = 5)	Paroxysmal AF (n = 10)	*p* value
**Demographics**			
Age, y	56 ± 1	61 ± 8	0.48
BMI (kg/m^2^)	26 [24–26]	26 [24–29]	0.46
HR (beats/min)	66 ± 7	59 ± 6	0.05
**Stroke risk factors (no, %)**			
CHF/LV dysfunction	0	0	NA
Hypertension	1 (20%)	2 (20%)	1.00
Age 65–74	2 (40%)	3 (30%)	0.69
Diabetes mellitus	1 (20%)	0	0.33
Stroke/TIA	0	0	NA
Vascular disease	0	0	NA
Aged 75 or over	0	0	NA
Female	1 (20%)	3 (30%)	1.0
**Cardioversion history**			
Previous CV (no, %)	NA	5 (50%)	NA
Duration since CV (m)	NA	24 [22–35]	NA
**Thromboembolism prophylaxis**			
ASA (no, %)	0	0	NA
VKA (no, %)	0	2 (20%)	0.52
NOAC (no, %)	0	5 (50%)	0.10

Abbreviations: *AF* atrial fibrillation, *BMI* body mass index, *HR* hear rate, *CHF* chronic heart failure, *LV* left ventricle, *TIA* transient ischemic attack, *CV* cardioversion, *m* months, *ASA* acetylsalicylic acid, *VKA* vitamin K-antagonist, *NOAC* novel oral anticoagulants.

### Left atrial function

The left atrial and left ventricular volume and function features of the subjects are listed in Table 3. No significant differences were detected for LA volumes and functional characteristics between the groups. Furthermore, LA global longitudinal strain and strain rates demonstrated comparable results between the groups (Table 4). The possible influence of LV function on LA longitudinal strain was assessed by analysis of LV longitudinal strain. Consistent with LA strain, LV longitudinal strain was comparable between the groups, as well.

**Table 3:** Left atrial and left ventricular volumes and function.

	Controls (n = 5)	Paroxysmal AF (n = 10)	*p* value
**LA volume indexed (mL/m**^**2**^**)**			
Maximum	36.6 ± 6.7	39.6 ± 10.4	0.57
Before atrial contraction	26.8 ± 6.4	29.5 ± 8.4	0.55
Minimum	15.8 ± 5.6	19.9 ± 7.0	0.27
Reservoir	20.8 ± 3.5	19.6 ± 5.1	0.65
Conduit	9.8 ± 2.4	10.0 ± 3.2	0.86
Active emptying	11.1 ± 2.6	9.6 ± 3.5	0.41
**LA function (%)**			
Total	60.8 [50.7–62.9]	52.8 [47.3–56.1]	0.50
Passive	27.1 ± 7.3	25.8 ± 6.4	0.71
Booster	42.2 ± 8.4	33.0 ± 9.2	0.08
**Left ventricle**			
EDV (mL)	173.4 ± 47.0	177.4 ± 48.1	0.88
ESV (mL)	61.8 ± 19.3	68.4 ± 28.7	0.65
SV (mL)	111.6 ± 29.3	106.0 ± 18.1	0.65
EF (%)	66.7 ± 2.7	64.0 ± 6.8	0.42
Mass (g)	118.5 ± 40.6	122.4 ± 27.8	0.82

Abbreviations: *AF* atrial fibrillation, *LA* left atrial, *mL* milliliter, *EDV* end-diastolic volume, *ESV* end-systolic volume, *SV* stroke volume, *EF* ejection fraction, *gr* gram.

**Table 4:** Left atrial and left ventricular strain.

	Controls (n = 5)	Paroxysmal AF (n = 10)	*p* value
**LA global longitudinal strain (%)**			
Reservoir	20.8 [14.3–22.4]	15.8 [14.7–16.8]	0.15
Conduit (–)	13.1 [14.9–7.4]	9.5 [10.6–8.3]	0.29
Active emptying (–)	6.8 [9.0–5.9]	6.1 [6.9–5.8]	0.42
**LA global longitudinal strain rate (s**^**−1**^**)**			
Peak positive	0.80 [0.65–0.90]	0.60 [0.50–0.72]	0.06
Peak early negative (–)	1.20 [1.55–0.80]	0.9 [1.20–0.57]	0.21
Peak late negative (–)	0.70 [1.20–0.65]	0.65 [0.72–0.60]	0.16
LV global longitudinal strain (%) (–)	18.39 ± 1.18	16.60 ± 2.04	0.09

Abbreviations: *AF* atrial fibrillation, *LA* left atrial, *LV* left ventricular.

### Left atrial flow

**Figure 3:**
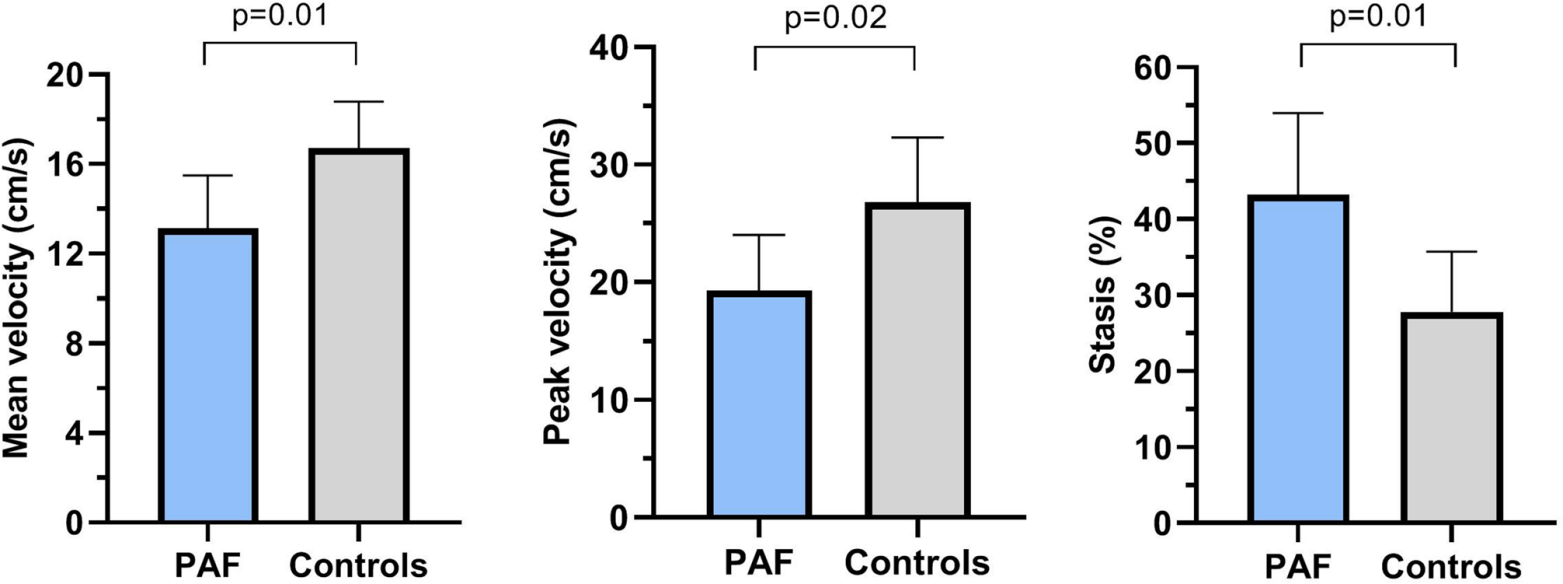
Comparison of mean flow velocity, peak flow velocity, and stasis fraction between patients with paroxysmal AF and controls. Data shown as mean and standard deviation. (GraphPad Prism, version 8.2.1, https://www.graphpad.com/scientific-software/prism/). Abbreviations: PAF, paroxysmal atrial fibrillation; cm/s, centimeter per second.

**Figure 4:**
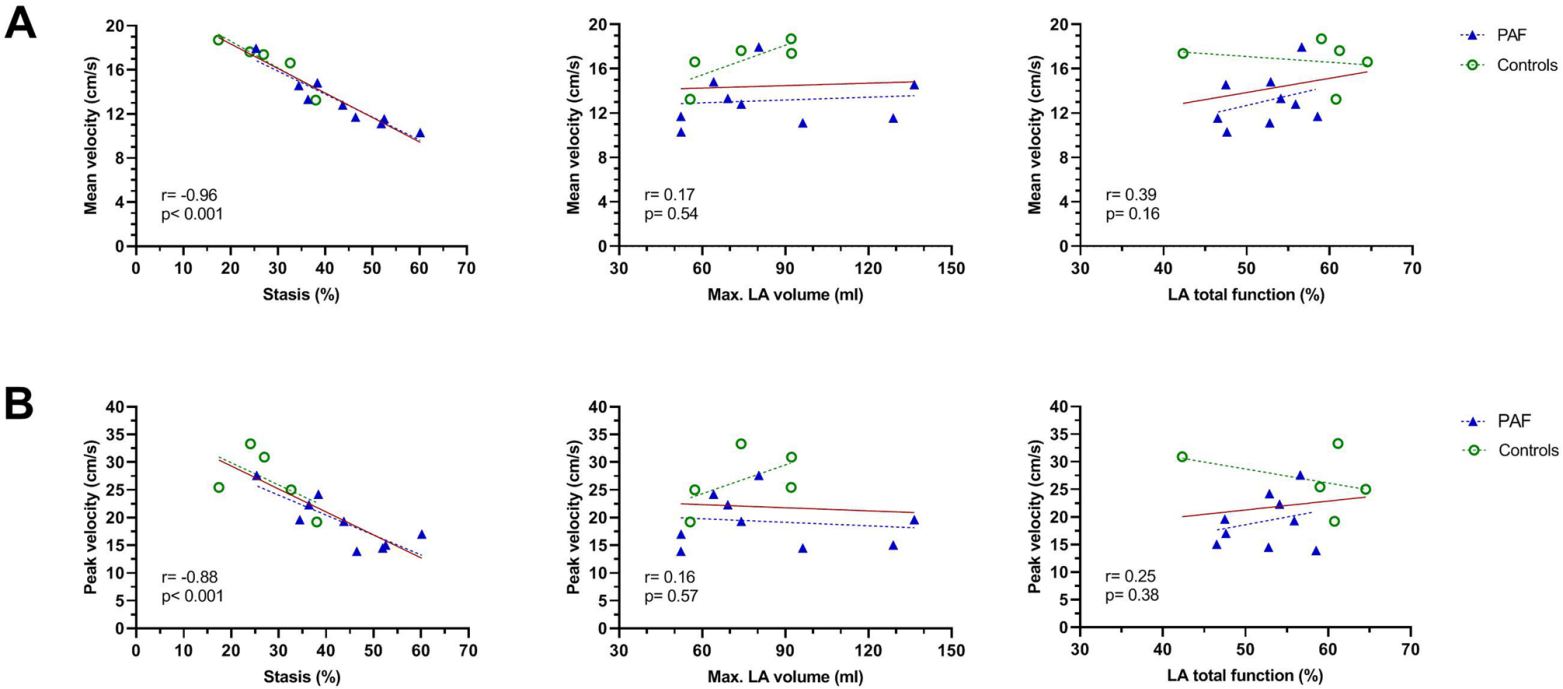
Relationship between mean flow velocity and stasis—maximum LA volume—LA total function (**A**) and relationship between peak flow velocity and stasis—maximum LA volume—LA total function (**B**) in the entire study cohort and each group (PAF and controls). The red line indicates the regression line for the entire cohort, whereas dashed lines are for each study group. (GraphPad Prism, version 8.2.1, https://www.graphpad.com/scientific-software/prism/). Abbreviations: Max, maximum; cm/s, centimeter per second; LA, left atrium; PAF (paroxysmal atrial fibrillation).

Figure 3 depicts the global mean flow velocity, peak velocity, and stasis fraction within the left atrium during the entire cardiac cycle for both groups. Even when in sinus rhythm, mean and peak LA flow velocities over the entire cardiac cycle were significantly lower in paroxysmal AF patients compared to controls [(13.1 ± 2.4 vs. 16.7 ± 2.1 cm/s, *p* = 0.01) and (19.3 ± 4.7 vs. 26.8 ± 5.5 cm/s, *p* = 0.02), respectively]. Moreover, paroxysmal AF patients expressed more stasis of blood than controls [43.2 ± 10.8% vs. 27.8 ± 7.9%, *p* = 0.01]. Table 5 provides a detailed assessment of the LA flow characteristics and the differences between the groups. Interestingly, LA mean and peak flow velocities were also lower in paroxysmal AF patients than controls during systole and as well as diastole. Figure 4 demonstrates the relationship between LA mean/peak flow velocities and stasis, maximum LA volume, and total LA function. In the whole study cohort, mean and peak LA flow velocities obtained over the entire cardiac cycle demonstrated a significant inverse correlation with stasis [mean velocity: r =  − 0.96, *p* < 0.001, peak velocity r =  − 0.88, *p* < 0.001]. However, no correlation was observed between LA flow velocities and LA volumetric and functional measures. Similar correlation patterns and significance levels were found when evaluated within each study group (paroxysmal AF, controls).

**Table 5:** Left atrial flow characteristics.

	Controls (n = 5)	Paroxysmal AF (n = 9)	*p* value
**Flow velocity (cm/s)**			
Velocity mean (R-R interval)	16.7 ± 2.1	13.1 ± 2.4	0.01
Velocity peak (R-R interval)	26.8 ± 5.5	19.3 ± 4.7	0.02
Velocity mean (systole)	17.4 ± 3.3	13.3 ± 1.6	< 0.01
Velocity peak (systole)	22.1 ± 6.1	16.4 ± 3.2	0.04
Velocity mean (diastole)	16.6 [13.7–17.6]	12.1 [10.3—14.6]	0.05
Velocity peak (diastole)	22.2 [20.4–29.2]	17.0 [14.4—21.8]	0.05
Velocity E wave	23.7 ± 5.8	17.0 ± 5.7	0.06
Velocity A wave	12.1 ± 2.8	11.0 ± 3.3	0.51
Stasis (%)	27.8 ± 7.9	43.2 ± 10.8	0.01
**Kinetic energy (mJ)**			
KE mean (R-R interval)	0.95 [0.75–1.87]	0.74 [0.59–1.24]	0.31
KE peak (R-R interval)	2.78 [1.54–4.40]	1.48 [1.03–2.52]	0.12
KE mean (systole)	0.96 [0.68–2.40]	0.84 [0.60–1.13]	0.35
KE peak (systole)	1.85 [1.15–4.40]	1.26 [0.92–1.64]	0.12
KE mean (diastole)	0.98 [0.77–1.30]	0.64 [0.50–1.17]	0.31
KE peak (diastole)	2.32 ± 0.85	1.57 ± 0.78	0.12
KE E wave	2.25 ± 0.78	1.36 ± 0.92	0.09
KE A wave	0.29 [0.16–0.87]	0.34 [0.29–0.99]	0.46
**Kinetic energy indexed (uJ/ml)**			
KE mean (R-R interval)	16.0 ± 4.5	10.6 ± 3.4	0.02
KE peak (R-R interval)	37.5 ± 11.7	19.9 ± 9.5	0.01
KE mean (systole)	18.0 ± 7.8	11.1 ± 2.6	0.02
KE peak (systole)	32.4 [17.6–47.8]	16.47 [14.0–18.7]	0.03
KE mean (diastole)	13.3 [11.9–16.1]	8.8 [6.5–11.8]	0.04
KE peak (diastole)	30.6 [25.2–36.1]	18.5 [12.3–21.8]	0.03
KE E wave	29.8 ± 5.7	17.5 ± 9.8	0.02
KE A wave	5.9 ± 3.7	5.39 ± 3.1	0.78

Abbreviations: *AF* atrial fibrillation, *cm/s* centimeters per second, *KE* kinetic energy, *uJ/ml* microjoule/milliliter, *mJ* milijoule.

### Left atrial energy

**Figure 5:**
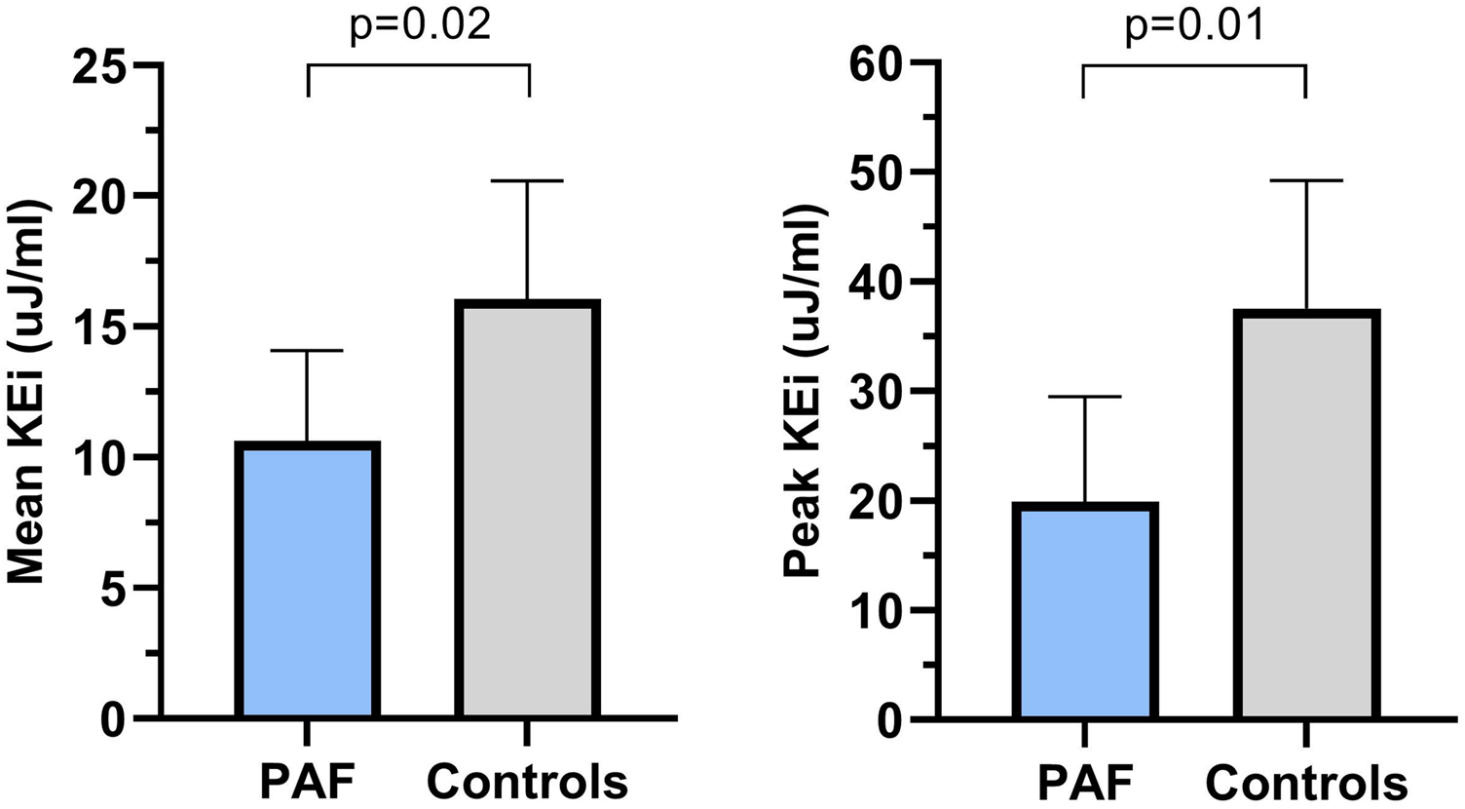
Comparison of mean and peak KE indexed to the maximum LA volume between patients with paroxysmal AF and controls. Data shown as mean and standard deviation. (GraphPad Prism, version 8.2.1, https://www.graphpad.com/scientific-software/prism/). Abbreviations: PAF, paroxysmal atrial fibrillation; KEI, kinetic energy indexed to maximum LA volume; uJ/ml, microjoule/milliliter; LA, left atrium.

Absolute KE values were lower in paroxysmal AF patients compared to controls, although the differences were not statistically significant (Table 5). Figure 5 reveals the KE values indexed to maximum LA volume over the entire cardiac cycle for both groups. The indexed mean and peak KE values were significantly lower in paroxysmal AF patients compared to controls [(10.6 ± 3.4 uJ/ml vs. 16.0 ± 4.5 uJ/ml, *p* = 0.02) and (19.9 ± 9.5 uJ/ml vs. 37.5 ± 11.7 uJ/ml, *p* = 0.01) respectively]. Furthermore, both indexed systolic and diastolic KE values were found to be significantly lower in patients with paroxysmal AF compared to the control group indicating the altered energy levels throughout the cardiac cycle.

### Left atrial appendage characteristics

LA appendage features of the subjects including volume, function, flow velocity, stasis, KE are detailed in the supplementary file. No significant differences were detected for LA appendage volumes and functional characteristics between paroxysmal AF patients and controls. The LA appendage mean and peak flow velocities during the entire cardiac cycle were lower and the stasis fraction was higher compared to the LA in the entire study cohort and within the study groups. No significant differences were found for mean and peak LA appendage flow velocities and KE values obtained within the entire cardiac cycle between the groups. Paroxysmal AF patients demonstrated more stasis of blood than controls in the LA appendage (73.3 ± 5.7% vs. 52.8 ± 16.2%, *p* = 0.04).

### Inter-observer variability

Overall, the inter-observer intra-class correlation coefficients demonstrated good to excellent agreement for LA 4D flow and feature tracking strain data. Intra-class correlation coefficients were 0.97 (95% CI 0.93–0.99) for LA mean flow velocity, 0.95 (95% CI 0.87–0.98) for LA peak flow velocity, both over the entire cardiac cycle, and 0.93 (95% CI 0.72–0.98) for stasis.

## Discussion

In the present study, we described 4D flow CMR-derived LA flow characteristics including flow velocity, stasis fraction, and kinetic energy in patients with paroxysmal AF and controls. Mean and peak flow velocities in the left atrium over the whole cardiac cycle were significantly lower and stasis fraction was higher in patients with paroxysmal AF compared to age/gender matched controls, even during sinus rhythm. Secondly, stasis fraction in the LA appendage was significantly higher in the paroxysmal AF patients than in controls. Thirdly, kinetic energy values indexed to maximum LA volume revealed markedly lower values in patients with paroxysmal AF compared to controls. Finally, no significant differences were observed between the groups for LA volume and function parameters including LA strain and strain rate profiles. The reduced global LA flow characteristics and increased stasis as determined by 4D flow analysis, therefore, may be of important clinical relevance to identify patients prone to thromboembolic complications, even in the absence of actual AF or changed LA geometry or function.

Although the Virchow’s triad was proposed more than a century ago, it is still widely accepted and comprehensively defines the main factors leading to thrombogenesis^18^. These factors are: abnormal blood stasis or reduced flow, endothelial/endocardial injury or dysfunction, and hypercoagulability. Yet, stroke risk stratification models (e.g., CHA_2_DS_2_-VASc) for patients with AF currently mostly rely on demographic and clinical scores and less on LA remodeling factors or flow characteristics. Of note, the accuracy of these risk scoring systems to predict ischemic stroke is only modest (C statistic∼0.6)^19,20^. This may result in overuse of anticoagulation in low-risk patients or might leave some individuals unprotected against thromboembolism who should require anticoagulation.

A comprehensive evaluation of flow velocities and stasis within the left atrium applying advanced imaging techniques may provide enhanced approaches for a robust assessment of the thromboembolism risk in patients with paroxysmal AF. 4D flow CMR potentially enables to improve risk assessment, providing global flow velocity characterization including stasis fraction within the left atrium. Using this technique, we have found that all mean and peak flow velocities determined over the whole cardiac cycle in the left atrium were significantly lower in patients with paroxysmal AF and stasis fraction was higher compared to age/gender matched controls. Importantly, these results appear to be independent of the blood viscosity factors (e.g., hematocrit levels), as they were comparable between the study groups. These findings suggest that global flow velocity parameters in the left atrium are remarkably impaired even during sinus rhythm in patients with paroxysmal AF. Conversely, Fluckiger et al. reported comparable mean flow velocity values in the left atrium between patients with paroxysmal AF and controls in a feasibility study^14^. However, only 6 patients with paroxysmal AF were included in this study and all these patients were male. In a recent larger study, Lee et al. documented lower mean and peak flow velocity values in the left atrium in paroxysmal AF patients compared to controls, which is in accordance with our results^6^. Importantly, this study cohort included subjects with mitral regurgitation and subjects with a short time period (min. 12 days) from the last rhythm control procedure, which may affect the status of global flow velocities in the left atrium. Of note, in these 2 reported studies, segmentation of the left atrium was performed through 4D flow data itself. In our study, we co-registered high spatial and temporal resolution b-SSFP cine images with 3-dimensional and 3-directional flow images. This provided a more precise segmentation at the cost of increased acquisition time. On the other hand, some studies observed a significant inverse relation between LA mean/peak flow velocities and CHA_2_DS_2_-VASc risk-score in patients with AF^6,21^. Overall, these findings provide evidence regarding altered LA flow dynamics in patients with paroxysmal AF indicating the importance of evaluating global LA flow velocities.

In the current study, a detailed assessment of LA remodeling parameters was performed with an acquisition protocol covering the entire left atrium and additional feature tracking strain analysis. Although main LA volume parameters were higher and total LA function was lower in absolute numbers in patients with paroxysmal AF than controls, the differences were not statistically significant. These results do not entirely comply with the accumulated clinical evidence, which reveal that AF leads to enlargement and dysfunction of the left atrium over time^9–11^. The most likely explanation may be that the sample size of the study was not large enough to provide significant differences or patients with large LA volumes were not included in the study, since the patients were in the workup for pulmonary vein isolation. Of note, as mentioned above, the study groups did demonstrate clear differences in terms of flow characteristics including mean/peak flow velocity and stasis fraction in the left atrium. LA anatomy and function may therefore provide incomplete information for thromboembolic risk assessment and we speculate that flow characteristics may deteriorate in the left atrium before structural and functional changes become evident in paroxysmal AF patients.

An important part of the total work of the heart transfers into kinetic energy (KE) of blood and KE is directly involved in the movement of blood^22,23^. Quantification of KE in the left atrium may therefore provide novel insights into intra-cardiac hemodynamics and may enhance understanding of blood flow stasis in patients with AF. This quantification of KE simply relies on blood density (fixed value), mass, and flow velocity (KE = ½ mv^2^). Although paroxysmal AF patients demonstrated overall reduced flow velocities in the left atrium, no significant difference was found regarding KE values during the cardiac cycle between the groups. This may be explained by small differences in LA volumes between the groups, as LA volumes (max, before contraction, min) were higher in paroxysmal AF patients. Nevertheless, overall absolute KE values were found lower in paroxysmal AF patients than controls. Through indexing KE values to the maximum LA volume, a KE density measure proportional to the squared velocity can be obtained. A recent study presented different volume-indexed KE results between the atria in healthy individuals signifying the determinant role of velocity in LA energetics^23^. In our study, we found that indexed KE values were significantly lower over the entire cardiac cycle compared to controls. Consequently, these results indicate that patients with paroxysmal AF might have altered energy levels in the left atrium, most likely because of impaired flow dynamics. Moreover, we observed that instant flow velocity and indexed KE values during the E wave were significantly different between the groups, but not during the A wave. This implies that, although restoration of sinus rhythm could provide comparable instantaneous flow dynamics during A wave, impaired flow hemodynamics can still persist throughout the cardiac cycle. LA energetics are directly associated with the efficiency of filling and pumping function of the left atrium and represent a novel alternative quantitative measure of LA flow, which may potentially have a role in thrombus formation. In addition, altered local energy levels in the left atrium may contribute to further remodeling of the left atrium occurring due to AF through associated changes in the pressure gradient^24^. Future studies focusing on kinetics and the atrial energy level are needed to clarify the role of KE in AF-related pathologies affecting left atrium hemodynamics, including flow stasis.

Given that thrombus has a predilection to arise in the LA appendage, we performed a sub-analysis for LA appendage flow characteristics. In general, LA appendage mean and peak flow velocities appeared to be lower and stasis fraction to be higher than in the LA. No significant differences for mean and peak LA appendage flow velocities were observed between the paroxysmal AF patients and controls, which are in line with previous results^21^. In addition, we observed no differences for LA appendage energetics between the groups. Although no differences were found for flow velocity and KE, stasis fraction was found to be noticeably higher in paroxysmal AF patients than in controls. This may be partially explained by the fact that the stasis fraction gives more detailed information on stagnant flows in the region of interest, as it indicates a proportion of the flow velocities. These findings suggest that stasis fraction may be a more sensitive indicator of the altered flow characteristics both within the LA and LA appendage for AF.

### Clinical implications and future directions

Several gaps in knowledge persist concerning the prediction of stroke and accurate anticoagulation management in patients with paroxysmal AF. Relevant studies have been limited by neglecting flow characteristics within the left atrium. Following technical advancements, 4D flow analysis allows for an accurate and comprehensive flow analysis within the left atrium. This study provides important novel insights by demonstrating impaired flow dynamics in the left atrium in patients with paroxysmal AF, even during sinus rhythm. It is yet unknown whether these changes in flow characteristics may be used as an early biomarker for stroke risk assessment, but these findings may indicate a potential attributing factor behind the occurrence of stroke in the absence of actual AF. Future large prospective studies are warranted to establish the role of LA flow in identifying patients with paroxysmal AF who will benefit from anticoagulation therapy.

### Limitations

This study has some limitations that need to be discussed. The sample size of the study (n = 15) is small and significantly limits the ability to draw firm clinical conclusions and this study should be considered hypothesis-generating rather than confirmative. However, the study was planned as an explorative study to evaluate essential details regarding flow characterization of the left atrium in patients with paroxysmal AF, which succeeded. Another limitation is related to the prospective acquisition of 4D flow images, which led to covering approximately 90% of the R-R interval. Nevertheless, b-SSFP cine images were retrospectively acquired covering the entire R-R interval and co-registered cine and 4D flow images were analyzed using temporally equal intervals, optimizing the reliability of the data. Our results regarding LA appendage flow parameters should be assessed with caution, as a high level of noise and the inherent low spatial resolution may have affected the results. Finally, the acquisition and post-processing time of the 4D flow data were long (e.g., > 15 min. and > 90 min, respectively).

## Conclusion

Assessment of global LA flow characteristics using 4D flow CMR in patients with paroxysmal AF presented impaired flow dynamics compared to controls without cardiac diseases, despite no obvious significant differences in LA volume or function. This pathophysiological flow pattern may explain the increased incidence of thromboembolic events in paroxysmal AF patients, even during sinus rhythm. Future large-scale studies are warranted to assess whether individual flow characteristics using 4D flow CMR may provide additional clinical value in stroke risk assessment and guidance of anticoagulation therapy.

## Supplementary Information

Supplementary Information

## Data Availability

The datasets generated during and/or analyzed during the current study are available from the corresponding author on reasonable request.
