# Supplementary material for: Altered left atrial 4D flow characteristics in patients with paroxysmal atrial fibrillation in the absence of apparent remodeling
Source: Sci Rep. 2021 Mar 16;11:5965. doi: 10.1038/s41598-021-85176-8 (PMC7966746; doi:10.1038/s41598-021-85176-8)
Supplement: Supplementary file 1 — Supplementary Information [file 41598_2021_85176_MOESM1_ESM.docx]

**Altered left atrial 4D flow characteristics in patients with paroxysmal atrial fibrillation in the absence of apparent remodeling**

Ahmet Demirkiran^1^ MD, Raquel P. Amier^1^ MD, Mark BM Hofman^2^ PhD, Rob J. van der Geest^3^ PhD, Lourens F.H.J. Robbers^1^ MD PhD, Luuk H.G.A. Hopman^1^ Msc, Mark J. Mulder^1^ MD, Peter van de Ven^4^ PhD, Cornelis P. Allaart^1^ MD PhD, Albert C. van Rossum^1^ MD PhD, Marco J. W. Götte^1*^ MD PhD, Robin Nijveldt^1,5*^ MD PhD

* *Both authors contributed equally*

***Affiliations:***

^1^ Department of Cardiology, Amsterdam UMC, Vrije Universiteit Amsterdam, Amsterdam Cardiovascular Sciences, Amsterdam, The Netherlands

^2^ Department of Radiology and Nuclear Medicine, Amsterdam UMC, Vrije Universiteit Amsterdam, Amsterdam, The Netherlands

^3^ Department of Radiology, Division of Image Processing, Leiden University Medical Center, Leiden, The Netherlands

^4^  Department of Epidemiology and Biostatistics, Amsterdam UMC, Vrije Universiteit Amsterdam, Amsterdam, The Netherlands

^5^ Department of Cardiology, Radboud University Medical Center, Nijmegen, The Netherlands

**Supplements**

- **Comparison of LA and LA appendage flow velocities and stasis within the entire study cohort, paroxysmal AF patients, and controls**
- **LA appendage volume and function characteristics**
- **Comparison of LA appendage flow characteristics between paroxysmal AF patients and controls**
- **Presentation of laboratory findings that may affect blood viscosity**

**Table 1** Left atrial and left atrial appendage flow characteristics (R-R interval)

|  | LA | LA appendage | P value |
| --- | --- | --- | --- |
| Entire study cohort |  | | |
| Velocity mean (cm/s) | 14.6±2.8 | 10.3±2.8 | <0.001 |
| Velocity peak (cm/s) | 22.0±6.1 | 18.2±5.9 | 0.08 |
| Stasis (%) | 37.1±12.4 | 65.4±14.6 | <0.001 |
| Paroxysmal AF patients |  | | |
| Velocity mean (cm/s) | 13.1±2.4 | 10.2±2.7 | <0.001 |
| Velocity peak (cm/s) | 19.3±4.7 | 18.8±6.6 | 0.75 |
| Stasis (%) | 43.2±10.8 | 73.3±5.7 | <0.001 |
| Controls |  | | |
| Velocity mean (cm/s) | 16.7±2.1 | 10.4±3.2 | 0.01 |
| Velocity peak (cm/s) | 26.8±5.5 | 17.2±5.0 | 0.06 |
| Stasis (%) | 27.8±7.9 | 52.8±16.2 | 0.01 |

Abbreviations: *AF, atrial fibrillation; cm/s, centimeters per second; LA, left atrial.*

**Table 2** Left atrial appendage volume (indexed) and function characteristics

|  | Controls | Paroxysmal AF | P value |
| --- | --- | --- | --- |
| EDV (mL/m^2^) | 1.62±1.02 | 2.34±1.61 | 0.38 |
| ESV (mL/m^2^) | 0.30±0.21 | 1.01±1.30 | 0.14 |
| SV (mL/m^2^) | 1.40±1.09 | 1.27±0.46 | 0.81 |
| EF (%) | 36.98±12.09 | 31.70±11.88 | 0.44 |

Abbreviations: *AF, atrial fibrillation; cm/s, centimeters per second; EDV, end-diastolic volume; ESV, end-systolic volume; SV, stroke volume; EF, ejection fraction.*

**Table 3** Left atrial appendage flow characteristics

|  | Controls | Paroxysmal AF | P value |
| --- | --- | --- | --- |
| Flow velocity (cm/s) |  | | |
| Velocity mean (R-R interval) | 10.5±3.3 | 10.2±2.7 | 0.88 |
| Velocity peak (R-R interval) | 17.2±5.0 | 19.7±6.4 | 0.46 |
| Velocity mean (systole) | 11.4±3.4 | 10.0±1.7 | 0.33 |
| Velocity peak (systole) | 17.0±5.0 | 17.0±4.6 | 0.99 |
| Velocity mean (diastole) | 9.6±3.5 | 10.4±3.5 | 0.69 |
| Velocity peak (diastole) | 13.1±4.7 | 17.6±7.8 | 0.27 |
| Velocity E wave | 12.2±4.5 | 14.1±7.3 | 0.60 |
| Velocity A wave | 9.9±4.6 | 12.8±7.4 | 0.45 |
| Stasis (%) | 52.8±16.2 | 73.3±5.7 | 0.04 |
| Kinetic energy (mJ) |  | | |
| KE mean (R-R interval) | 0.03±0.01 | 0.04±0.02 | 0.78 |
| KE peak (R-R interval) | 0.08±0.03 | 0.09±0.02 | 0.39 |
| KE mean (systole) | 0.03±0.01 | 0.03±0.02 | 0.91 |
| KE peak (systole) | 0.06±0.04 | 0.08±0.02 | 0.48 |
| KE mean (diastole) | 0.02±0.01 | 0.03±0.02 | 0.59 |
| KE peak (diastole) | 0.05±0.03 | 0.07±0.04 | 0.29 |
| KE E wave | 0.04±0.03 | 0.05±0.03 | 0.71 |
| KE A wave | 0.02±0.02 | 0.04±0.04 | 0.39 |
| Kinetic energy indexed (uJ/ml) |  | | |
| KE mean (R-R interval) | 15.1±7.7 | 8.4±3.1 | 0.12 |
| KE peak (R-R interval) | 32.4±16.2 | 22.2±9.5 | 0.17 |
| KE mean (systole) | 17.9±10.0 | 8.6±2.6 | 0.10 |
| KE peak (systole) | 30.6±18.5 | 20.4±8.9 | 0.20 |
| KE mean (diastole) | 12.1±6.1 | 14.6±18.0 | 0.77 |
| KE peak (diastole) | 19.7±6.7 | 16.9±9.7 | 0.58 |
| KE E wave | 17.5±5.0 | 13.7±11.5 | 0.51 |
| KE A wave | 9.2±10.0 | 7.8±9.9 | 0.80 |

Abbreviations: *AF, atrial fibrillation; cm/s, centimeters per second; KE, kinetic energy; uJ/ml, microjoule/milliliter; mJ, milijoule.*

**Table 4** Laboratory findings

|  | Controls | Paroxysmal AF | P value |
| --- | --- | --- | --- |
| Cholesterol (mmol/l) | 4.85±0.72 | 4.98±0.77 | 0.77 |
| High-density lipoprotein (mmol/l) | 1.38±0.30 | 1.23±0.40 | 0.24 |
| Low density lipoprotein (mmol/l) | 3.02±0.64 | 2.35±1.03 | 0.52 |
| Triglycerides (mmol/l) | 1.30 [0.77 – 1.67] | 1.20 [0.95 – 4.70] | 0.46 |
| Erythrocytes (x10E12/l) | 5.02±0.19 | 4.78±0.57 | 0.45 |
| Mean corpuscular volume (fl) | 90±2 | 89±6 | 0.75 |
| Hemoglobin (mmol/l) | 9.38±0.83 | 9.29±0.86 | 0.85 |
| Hematocrit (%) | 44±3 | 43±3 | 0.85 |
| Platelet (x10E9/l) | 265±64 | 261±44 | 0.88 |
| Activated partial thromboplastin time (sec) | 27.25±4.03 | 37.20±11.91 | 0.15 |
| International normalized ratio | 1.07 [1.01 – 1.10] | 1.27 [1.06 – 1.70] | 0.25 |

Abbreviations: *AF, atrial fibrillation.*
